# A vascular endothelial cell, neuron, and microglia tri-culture model to study hypertension-related depression

**DOI:** 10.3389/fncel.2025.1553309

**Published:** 2025-03-31

**Authors:** Hongxia Zhao, Lingge Huang, Jian Liu, Min Feng, Yeqian Liu, Hong Li, Shan Gong, Chunming Chen, Shuiqing Zeng, Weiqiong Ren

**Affiliations:** ^1^Department of Pharmacy, The First Hospital of Hunan University of Traditional Chinese Medicine, Changsha, Hunan, China; ^2^The Second Clinical Medical College, Guangzhou University of Chinese Medicine, Guangzhou, Guangdong, China; ^3^Department of Pharmacy, The Second People’s Hospital of Anhui, Hefei, Anhui, China

**Keywords:** co-culture, hypertension, depression, inflammation, TLR4, NF-κB, neurotransmitters, hippocampal

## Abstract

Hypertension-related Depression (HD) is a complex mental disorder that exerts a significant negative impact on patients’ quality of life. Previous studies have demonstrated that damages to vascular endothelial and hippocampus are the primary pathological features in HD rats. Under hypertensive conditions, inflammatory cytokines in peripheral blood vessels can induce central nervous system inflammation through penetration of a damaged blood-brain barrier, peripheral immune cells, and neural pathways, damaging the brain and triggering HD. Therefore, interactions between vascular endothelial cells, neurons, and glial cells are critical for the understanding of HD. However, *in vivo* animal models are often limited by the complexity of intrinsic systems, high inter-individual variability, and stringent ethical regulations. A reliable model that could be easily manipulated is needed for investigating the mechanisms involved in communication between vascular endothelial cells, neurons, and glial cells in HD. We therefore aimed to create a composite tri-culture model consisting of rat aortic endothelial cells (RAECs), neurons, and microglia to study HD. First, RAECs were stimulated with lipopolysaccharide to mimic endothelial injury under hypertensive conditions. Vascular endothelial function and inflammatory levels were assessed using fluorescent probes and enzyme-linked immunosorbent assays. RAECs treated with 1 μg/ml LPS for 24 h had reduced levels of nitric oxide, increased levels of endothelin-1 and inflammatory mediators. These findings are consistent with the endothelial dysfunction and inflammatory responses observed in spontaneously hypertensive rats, which suggests that the lipopolysaccharide-induced RAECs model effectively mimics key pathological features of hypertension-related endothelial injury. Subsequently, the supernatants from lipopolysaccharide-induced RAECs were combined with 200 μM corticosterone and transferred to neuron-microglia co-cultures to simulate damages to hippocampal neuron under HD conditions. To evaluate the features of cells, neuronal viability was measured by CCK-8 and live-dead assays. Nissl staining was used to assess neuronal Nissl bodies, while the levels of inflammatory factors and monoamine neurotransmitters in the culture supernatants were evaluated by enzyme-linked immunosorbent assays. Reactive oxygen species in neurons were visualized by a fluorescent probe, apoptosis was detected using TUNEL assays, and immunofluorescence was used to assess microglial phenotypes and the levels of TLR4 and NF-κB. It was found that neurons in the tri-culture model had reduced viability, higher levels of apoptosis, fewer Nissl bodies, increased inflammation, and reduced levels of monoamine neurotransmitters. Additionally, the number of M1 microglia was increased, along with elevated levels of TLR4 and NF-κB proteins. These findings were similar to damages of hippocampal neuron, abnormal levels of monoamine neurotransmitters, microglia polarization, and hippocampal inflammatory response observed in the HD rat model. In conclusion, our findings indicate that the tri-culture model can effectively simulate the pathological characteristics of HD, especially in vascular endothelial damage, neuroinflammation, monoamine neurotransmitters disorders. Therefore, the tri-culture model would provides a reliable and invaluable experimental tool for further research on the pathogenesis and treatment of HD.

## 1 Introduction

Hypertension affected approximately 1.3 billion adults globally in 2019 (Zhou et al., 2021), and the number is predicted to soar to 1.56 billion by 2025 (Forouzanfar et al., 2017). Depression, a common complication associated with hypertension, aggravates blood pressure and even mortality risk in patients with hypertension, which may affect the quality of life of people with hypertension-related depression (HD) (Li et al., 2015; Rantanen et al., 2018; Gan et al., 2023; Qiu et al., 2024). Although the combined use of antihypertensive and antidepressant therapies has shown efficacy (Gan et al., 2023), the pathogenesis of HD remains complex and multifactorial. Emerging evidence highlights the critical imbalances in the inflammatory response (Meng et al., 2020), monoamine neurotransmitter disorders (Li et al., 2024), and hippocampal atrophy (Zhao et al., 2021; Lee et al., 2022) during the progression of HD. The hippocampus is involved in the regulation of cognitive functions and emotion, and damage to hippocampal neurons is a key factor leading to depression (Zhang et al., 2022). However, the damage to these neurons is due to multiple factors. Specifically, inflammatory cytokines in the peripheral blood vessels can penetrate into the brain through the compromised blood-brain barrier, and the subsequent activation of glial cells will release additional proinflammatory cytokines, resulting in neuronal damage (Block et al., 2007; Cattaneo et al., 2015; Köhler et al., 2016). The elucidation of the intercellular communication mechanisms between vascular endothelial cells, neurons, and glial cells is crucial for the understanding of the underlying pathways by which hypertension triggers depression.

Depressive-like disorders are associated with the activation of glial cells and reduced cellular density and function in the central nervous system (Rial et al., 2016). Activated microglia have been found to regulate neuronal function in neuroinflammation triggered by stress through the secretion of proinflammatory cytokines and metabolites, suggesting their involvement in the development of depression (Jia et al., 2021; Zheng et al., 2021). The targeted elimination of microglia significantly reduces both neuroinflammation and blood pressure in spontaneous hypertensive rats (Shen et al., 2015). A previous study has shown that the exosomal microRNA, miR-9-5p, secreted by neurons promoted polarization of microglia to the M1 phenotype and aggravated depressive-like behavior and neuroinflammation in animal models of depression (Xian et al., 2022). Our previous studies observed that rats with HD showed increased levels of inflammation, activated M1-type microglia, and structural damage to neurons; it was found that both their tail artery pressure and depressive-like behavior were relieved by alleviating neuroinflammation (Ma et al., 2024). Therefore, a targeted study of the dynamic relationship between microglia and neurons will contribute to a better understanding of the mechanisms underlying neuroinflammatory diseases.

To our knowledge, there are two available rat animal models for studying the neuropathology of HD (Yang et al., 2023; Zhao et al., 2024). The HD rat model exhibits several features that closely resemble those observed in hypertensive patients with depression. Specifically, HD rats display higher blood pressure levels (above 130 mmHg) and depression-like behaviors such as behavioral despair, lack of enthusiasm, and other symptoms. Additionally, the levels of pro-inflammatory cytokines (TNF-α, IL-1β, and IL-6) are increased in HD rats. These features are consistent with the clinical manifestations of hypertensive patients with depression, who often present with elevated blood pressure, feelings of guilt and hopelessness, feeling blue, experiencing insomnia, and the inability to focus (Santoni et al., 2025). Moreover, the elevation of inflammatory markers such as hs-CRP is also a common feature in these patients (Wang et al., 2024). These models are reliable and commonly used in basic research as they resemble the human condition in terms of physiological conditions, genes, and pathogenesis (Lerman et al., 2019). However, the study of intercellular communication mechanisms is constrained by numerous factors inherent to animal models. Additionally, longer experimental times and greater costs are characteristic of animal models (Xie et al., 2016). While cell models for investigating hypertension or depression have been reported, models of endothelial dysfunction and neuroinflammation are relatively simple, rendering it difficult to replicate the complex pathogenic environment associated with HD (Zheng et al., 2021; Wang et al., 2023). Therefore, the establishment of an *in vitro* cell co-culture model that can simulate the HD environment is key to investigating HD.

In this study, we used conditioned media with a modification of the Banker co-culture method to establish and characterize a tri-culture model consisting of rat aortic endothelial cells, hippocampal neurons, and cortical microglia, and used this model to study the pathogenesis of HD.

## 2 Materials and methods

### 2.1 Rat aortic endothelial cells

Rat aortic endothelial cells (RAECs) (#CP-R075) were obtained from Pricella Biotechnology Co., Ltd., (Wuhan, China). The RAECs were cultured in a customized medium (Pricella, Wuhan, China,#CP-R075) at 37°C with 5% CO_2_, and were utilized for experiments during the first 10 passages.

### 2.2 Animals

Sprague-Dawley rats, including six pregnant rats (E16-18) and 24 neonatal rats (2–3 days old), were purchased from a laboratory animal supply company [Slac Jingda, Changsha, China; license no. SCXK (Xiang) 2019-0004]. All experiments were approved by the Ethical Committee of the First Hospital of Hunan University of Chinese Medicine (approval no. ZYFY20211029).

### 2.3 Evaluation of *in vitro* endothelial dysfunction models

#### 2.3.1 Detection of cell morphology and viability

RAECs (5 × 10^3^ per well) were seeded in 96-well plates and grown for 24 h before treatment with varying concentrations of lipopolysaccharide (LPS; 0, 0.01, 0.1, 1, 10 μg/mL) (Sigma-Aldrich, St Louis, MO, United States, #L2880) for 6, 12, 24, and 48 h. The morphology of the cells was evaluated using an inverted microscope (Mshot MSX2, Guangzhou, China) and the viability was assessed using CCK-8 assays (Elabscience, Wuhan, China, #E-CK-A362). After discarding the medium, 100 μL CCK-8 was added to each well, and they were incubated for 1 h at 37°C. Then, their absorbance (A) were detected at 450 nm using MK-3 microplate reader (Thermo Fisher, Waltham, MA, United States). The percentage of viability in the CCK-8 assay was calculated by the following equation:


$$\mathrm{Viability}\left(\%\right)=\frac{\text{Asample}-A\,\text{blank }}{\text{Acontrol}-A\,\text{blank }}\times 100.$$

#### 2.3.2 Detection of intracellular nitric oxide

After treatment, the RAECs were washed with phosphate-buffered saline (PBS), followed by the addition of 60 μM 1,2-Diaminoanthraquinone (MedChemExpress, Monmouth Junction, NJ, United States, #HY-W013435; Ex/Em = 485/538 nm) and incubation for 40 min at 37°C. Residual fluorochrome was subsequently removed by washing with serum-free medium. The cells were then stained with 100 μg/mL Hoechst 33342 (Beyotime, Shanghai, China, #C1022; Ex/Em = 350/461 nm) for 15 min at room temperature. Images were observed in laser confocal microscope (LSM800, Zeiss, Germany) and the level of nitric oxide (NO) was analyzed with ZEN 3.4 (Zeiss, Germany). To quantify the expression levels of NO, the mean fluorescence intensity (MFI) within defined regions of interest was measured for each cell. The level of NO was calculated as follows:


$$\mathrm{MFI}\left(\%\right)=\frac{\mathrm{Sum}\,\mathrm{of}\,\mathrm{MFI}\,\mathrm{for}\,\mathrm{all}\,\mathrm{cells}}{\mathrm{Total}\,\mathrm{number}\,\mathrm{of}\,\mathrm{cells}}\times 100.$$

#### 2.3.3 Measurement of cytokine levels in the culture supernatants

RAECs were treated with LPS for 24 h. after which the culture supernatants were collected and centrifuged (3,000 rpm, 20 min, 4°C). The levels of endothelin-1 (ET-1), monocyte chemotactic protein 1 (MCP-1), vascular cell adhesion molecule 1 (VCAM-1), tumor necrosis factor-alpha (TNF-α), interleukin-1beta (IL-1β), and interleukin-6 (IL-6) were measured using ELISA kits (Feiya Biotechnology, Jiangsu, China, #FY3435-A, #FY2206-A, #FY21351-A, #FY3056-A, #FY2923-A, and #FY3066-A, respectively) according to the manufacturer’s instructions. The absorbance was measured at 450 nm with an MK-3 microplate reader. The levels of cytokines were calculated using standard curves.

#### 2.4 Preparation of conditioned media of the hypertensive environment

RAECs (1 × 10^6^ per well) were cultured in 6-well plates for 24 h after which they were divided into the blank media group (BM) and the conditioned media of the hypertensive environment group (CMHE). The former were cultured in normal medium, while the latter were treated with 1 μg/mL LPS for 24 h. Culture supernatants were collected separately, followed by centrifugation (4,000 rpm, 10 min, 4°C) and filtration of the supernatants through 0.22 μm membrane filters, followed by storage at −*80*°C for no longer than 1 month.

### 2.5 Preparation of cortical microglia

In this study, an important change was implemented in the preparation of cortical microglia. Before the seeding of cortical cells into 24-well plates, dots of heated paraffin were placed in a triangular manner on the bottom of the plate, creating physical divisions between the microglial feeder layer and the upper coverslips with neurons (Wasilewski et al., 2022). All primary cell cultures were conducted under strict sterile conditions. Primary microglia were obtained from the cortical lobes of neonatal rats, as previously described (Liu et al., 2018). After washing the pups with 75% ethanol and subsequent decapitation, the cerebrum was immersed in DMEM/F12 medium (Gibco, Waltham, MA, United States; #8120254) for a few seconds. The adjoining meninges were stripped and bleeding spots were detached using a stereomicroscope. The cortical lobes were chopped into 1 mm^3^ pieces, followed by digestion with 0.25% trypsin (Gibco, #25200056) at 37°C for 12 min, halting the reaction by the addition of microglial growth medium consisting of DMEM/F12, 10% fetal bovine serum (FBS) (Gibco, #10270-106), 1% penicillin-streptomycin (Solarbio, Beijing, China; #P1400). The supernatant was discarded after centrifugation at 1,200 rpm for 5 min, after which the cell pellets were resuspended in microglial growth medium, filtered through 40 μm cell strainers, seeded in cell culture flasks at a density of 3 × 10^6^ cells/mL, and incubated at 37°C for 15 min to isolate the microglia from other glial cells. The cell suspensions were then transferred to 24-well plates with dots of heated paraffin. Lastly, half of the culture supernatant was replaced with the same volume of fresh microglial growth medium every 3 days until the cells reached confluence.

### 2.6 Preparation of hippocampal neurons

Coverslips used for seeding neurons were pre-treated. Coverslips that fit 24-well plates were placed in each well, after which 100 μg/mL poly-L-lysine (Solarbio, #P8130) solution was added to the wells and incubated for 1 h at 37°C. The poly-L-lysine was then discarded and the coverslips were returned to the incubator for at least 4 h. Lastly, the coverslips were washed three times with PBS before seeding neurons.

Pregnant rats with embryos at the E16-18 gestational period were anesthetized with 3% sodium pentobarbital at a dose of 2 mL/kg. The embryos were removed and placed on ice. The hippocampus was isolated from the cerebrum, divided into 1 mm^3^ pieces, and digested with a combination of 0.25% trypsin and 0.2% collagenase I (Solarbio, #C8140) for 10 min at 37°C. The reaction was terminated by adding neuronal seeding medium consisting of DMEM/F12 with 10% FBS, 1% penicillin-streptomycin, and 1% B27 supplement (Gibco, #17504-044). The cells were resuspended, filtered through 40 μm cell strainers, and centrifuged at 1,000 rpm for 5 min. The lower-layer neurons were seeded on pre-coated coverslips at a density of 5.0 × 10^5^ cells/mL (800 μL/well) and incubated for 2–4 h until adherent. The culture supernatant was then replaced with neuronal maintenance medium consisting of neurobasal medium (Gibco, #21103-049), 2% B27 Supplement, 1% Glutamax (Gibco, #35050061), 1% penicillin-streptomycin. Half of the culture supernatant was replaced with the same volume of fresh neuronal maintenance medium every 3 days.

### 2.7 Generation of the tri-culture system

**FIGURE 1:**
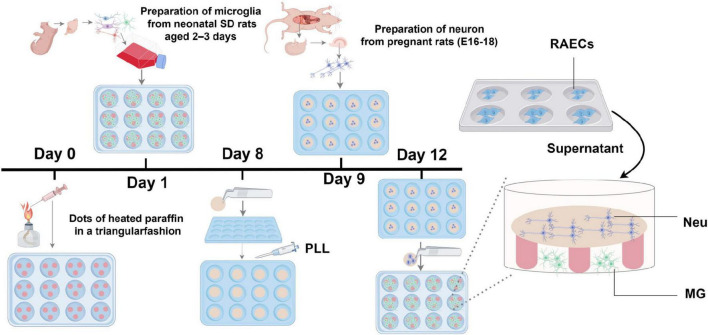
Diagram illustraing the *in vitro* tri-culture system. PLL, poly-L-lysine; Neu, hippocampal neuron; MG, cortical microglia.

To generate a tri-culture system for investigating intercellular communication in HD, it consisted of vascular endothelial cells, neurons, and microglia was constructed using a combination of conditioned medium and Banker’s co-culture technique (Kaech and Banker, 2006). Specifically, after culture of the microglia and neurons for 12–15 and 3–5 days, respectively, coverslips seeded with neurons were inverted over the paraffin dots and the microglial culture supernatant, creating a microenvironment enabling the concentration of factors secreted by the neurons and microglia for better cellular differentiation. The medium of the co-culture system was predominantly neuronal serum-free medium, with the microglial medium accounting for one-eighth of the overall volume of the culture medium. Since the microglial medium contains FBS, an excessive amount of it will lead to aggregation and death of the neurons. Subsequently, specific volumes of culture supernatants obtained from RAECs were added to neuron-microglia co-culture systems for 1–2 days to form a tri-culture system consisting of rat vascular endothelial cells, neurons, and microglia (Figure 1).

### 2.8 Modeling of the tri-culture system under HD conditions and pharmacological intervention *in vitro*

Hypertension-related depression conditions were simulated using the conditions identified in the preliminary experiments. Briefly, 10% CMHE combined with 200 μM corticosterone (CORT) (MedChemExpress, #HY-B1618) were added to the neuron-microglia co-culture system for 24 h. Then, 10 nM fluoxetine (Aladdin, Shanghai, China, #F189157) or 10 μM TAK-242 (MedChemExpress, #HY-11109) was added to the wells to assess cell viability, Nissl bodies, inflammation, monoamine neurotransmitters, and reactive oxygen species (ROS). The control groups consisted of 10% BM and single neurons.

### 2.9 Assessment of cell morphology

Cell morphology was assessed under inverted microscopy, and Nissl bodies in hippocampal neurons were evaluated using a Nissl staining solution (Beyotime, #C0117), as directed. Following microscopic imaging of the stained sections, qualitative assessment was conducted to evaluate neuronal integrity by analyzing the overall morphological characteristics of neurons, the staining intensity of Nissl bodies in both control and experimental groups.

### 2.10 Cell viability assay

The tri-culture system was incubated for 24 h with different treatments. Neurons were incubated with CCK-8 for 4 h at 37°C, after which the absorbance at 450 nm was detected using an MK-3 microplate reader and the cell viability was calculated by the equation in section “2.3.1 Detection of cell morphology and viability.”

### 2.11 Calcein-AM/PI live/dead cell double staining

Living and dead cells were stained, respectively by Calcein-AM (green, Ex/Em = 490/515 nm) and PI (red, Ex/Em = 535/617 nm) (Solarbio, #CA1630) following the guidelines provided by the manufacturer. Images were observed using a laser confocal microscope. After processing the images in ZEN software, positive cells of Calcein-AM and PI were quantified separately using ImageJ. The cell mortality rate (%) was calculated as follows:


Cellmortality(%)=

$$\frac{\mathrm{Number}\,\mathrm{of}\,\mathrm{PI}-\mathrm{positive}\,\mathrm{cells}}{\mathrm{Total}\,\mathrm{number}\,\mathrm{of}\,\mathrm{positive}\,\mathrm{cells}\,\left(\mathrm{Calcein}-\mathrm{AM}+\mathrm{PI}\right)}\times 100.$$

### 2.12 ELISA

The tri-culture system was cultured as described above. The culture supernatants were collected and centrifuged to remove cellular debris. Levels of IL-1β (AIDISHENG, Jiangsu, China, #ADS-R00040A) and TNF-α, IL-6, norepinephrine (NE), serotonin (5-HT), and dopamine (DA) (Feiya Biotechnology, #FY3056-A, #FY3066-A, #FY3643-A, #FY3318-A, and #FY3231-A, respectively) were measured by ELISA kits, as directed. The absorbance was measured at 450 nm using an MK-3 microplate reader. The levels of cytokines were calculated using standard curves.

### 2.13 Fluorescence probe assay

The procedure was the same as that used for the measurement of intracellular NO except that the fluorescence dye was 200 μM DCFH-DA (MedChemExpress, #HY-D0940; Ex/Em = 488/525 nm).

### 2.14 Immunofluorescence

After removal of the media, the cells were washed with PBS and incubated in 4% paraformaldehyde for 35 min, followed by permeabilization in 0.25% Triton X-100 for 10 min and blocking with 5% BSA for 15 min. The cells were then incubated for 12 h at 4°C with primary antibodies against MAP2 (1:100, Proteintech, Wuhan, China, #67015-1-Ig), Iba1 (1:400, Fujifilm-Wako, Japan, #019-19741), CD206 (1:200, Proteintech, #60143-1-Ig), CD16 (1:50, Proteintech, #16559-1-AP), TLR4 (1:200, Aifang Biological, Changsha, China, #AF11010), NF-κB (1:50, BOSTER, Wuhan, China, #A00284-1), Bcl-2 (1:200, Proteintech, #68103-1-Ig), and Bax (1:200, Proteintech, #50599-2-Ig). This was followed by incubation with CoraLite 594 or FITC fluorophore-conjugated secondary antibodies (1:200, Proteintech, #RGAR004, #SA00003-1) for 30 min at 37°C. Cell nuclei were counterstained with 5 μg/ml DAPI (Solarbio, #S2110, Ex/Em = 360/460) for 15 min at room temperature, and samples were analyzed with a laser confocal microscope. Fluorescence intensity was measured using ZEN or ImageJ software. To quantify the expression levels of TLR4, NF-κB, Bcl-2, and Bax proteins in neurons, as well as CD16 and CD206 proteins in microglia, MFI within defined regions was measured for each cell. The MFI of the target protein was calculated as follows:


$$\mathrm{MFI}\left(\%\right)=\frac{\mathrm{Sum}\,\mathrm{of}\,\mathrm{MFI}\,\mathrm{for}\,\mathrm{all}\,\mathrm{cells}}{\mathrm{Total}\,\mathrm{number}\,\mathrm{of}\,\mathrm{cells}}\times 100.$$

### 2.15 Apoptosis assay

Cells were carefully washed in pre-cooled PBS and were stained with One-Step TUNEL Apoptosis Kit (Elabscience, #E-CK-A322, Ex/Em = 590/617) according to the manufacturer’s instructions. Cells were subsequently stained with DAPI and images were observed using a laser confocal microscopy. Apoptotic cells were quantified by counting TUNEL-positive cells and normalizing to the total number of DAPI-stained nucleus. The cell apoptosis rate (%) was calculated as follows:


$$\mathrm{Apoptosis}\mathrm{rate}\left(\%\right)=\frac{\mathrm{Number}\,\mathrm{of}\,\mathrm{TUNEL}-\mathrm{positive}\,\mathrm{cells}}{\mathrm{Number}\,\mathrm{of}\,\mathrm{DAPI}-\mathrm{stained}\,\mathrm{nucleus}}\times 100.$$

### 2.16 Confocal microscopy and image processing

All fluorescence experiments were imaged using the laser confocal microscope (LSM800, Zeiss, Germany). Parameters including the excitation and emission wavelengths of the markers and exposure time were set, keeping them identical for imaging all groups. For each sample, three fields of view were randomly selected to acquire images, and the files were uniformly saved in the “czi” format. The images were subsequently analyzed using ZEN imaging software (Zeiss, version 3.4) or ImageJ software (version 1.53). The fluorescence threshold for markers was adjusted, and the same threshold was applied uniformly to all other images for consistency in ZEN software. MFI of markers was recorded, and images from individual channels as well as merged channels were saved in the “TIFF” format.

### 2.17 Statistical analysis

All experiments were performed independently at least three times (*n* ≥ 3). The data were analyzed with SPSS 27.0 software (IBM Corp., Armonk, NY, United States) and are presented as mean ± SD. Independent samples *t*-tests were used for comparisons between two groups, while one-way ANOVA followed by LSD or Tamhane’s T2 was used for multiple comparisons. Non-normally distributed data were analyzed using the Mann-Whitney U test or the Kruskal-Wallis test. Repeated-measures ANOVA was used for the statistical comparisons involving repeated measurements. Values of *P* < 0.05 were considered statistically significant.

## 3 Results

### 3.1 Dose- and time-dependent aggravation of injuries induced by LPS

**FIGURE 2:**
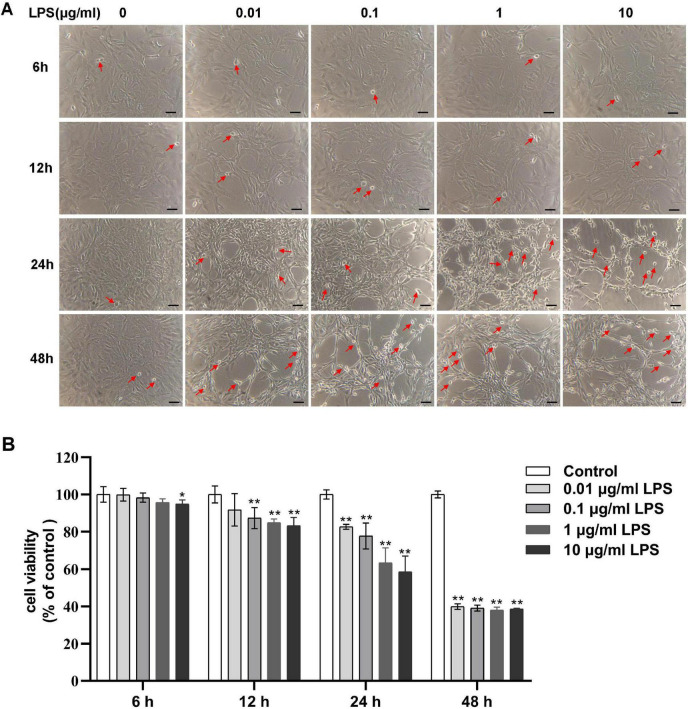
Concentration-dependent effects of LPS on the morphology and viability of RAECs. **(A)** Morphological changes in groups treated with 0, 0.01, 0.1, 1 and 10 μg/mL LPS for various times. Shrunken cells with high refractive index are marked with red arrows. **(B)** Concentration-dependent changes in cell viability in the different groups. Data are presented as the mean ± SD (*n* = 3). *P < 0.05, **P < 0.01 vs. control group (Repeated - measures ANOVA). Scale bar = 50 μm. LPS, lipopolysaccharide; RAECs, rat aortic endothelial cells.

**FIGURE 3:**
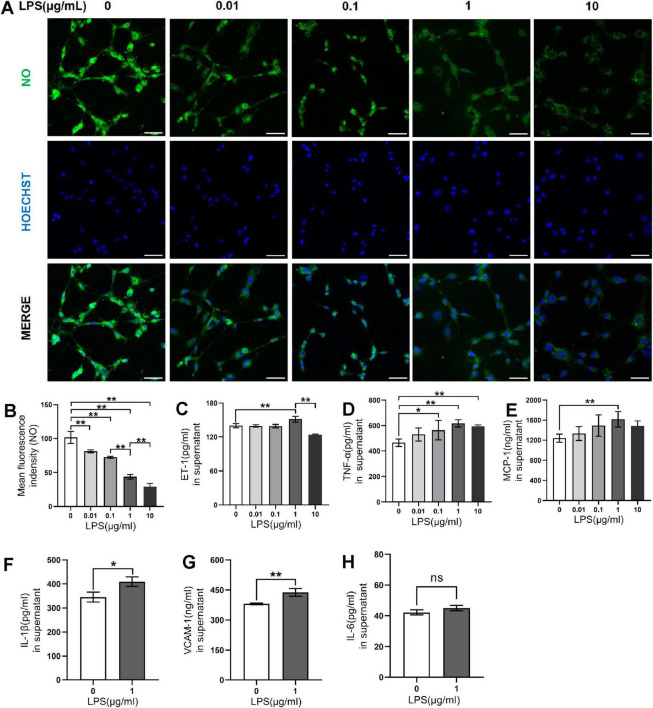
Effects of LPS on endothelial dysfunction and inflammation in RAECs. **(A)** Fluorescence images of NO in groups treated with 0, 0.01, 0.1, 1 and 10 μg/mL LPS for 24 h. **(B)** Changes in NO fluorescence intensity in the groups. **(C)** ET-1 levels in supernatant. **(D)** TNF-α levels in supernatant. **(E)** MCP-1 levels in supernatant. **(F)** IL-1β levels in supernatant. **(G)** VCAM-1 levels in supernatant. **(H)** IL-6 levels in supernatant. Data are presented as the mean ± SD (*n* = 3). *P < 0.05, **P < 0.01 vs. control group (one-way ANOVA followed by LSD or Tamhane’s T2 test, or *t*-tests for comparing two groups). Scale bar = 50 μm. LPS, lipopolysaccharide; RAECs, rat aortic endothelial cells; NO, nitric oxide; ET-1, endothelin-1; TNF-α, tumor necrsis factor alpha; MCP-1, monocyte chemotactic protein 1; IL-1β, interleukin-1beta; VCAM-1, vascular cell adhesion molecule 1; IL-6: interleukin-6.

To obtain the CMHE, RAECs were treated with 0, 0.01, 0.1, 1, and 10 μg/mL LPS for 6, 12, 24, and 48 h followed by observation of morphology. After treatment with LPS for 12 and 24 h, shrunken cells with high refractive index and intercellular spaces with significant increase were observed, indicating damage to cells (Figure 2A). Repeated Measures ANOVA was employed to evaluate the interactive effects of LPS concentration (0, 0.01, 0.1, 1, 10 μg/mL) and intervention durations (6, 12, 24, 48 h) on cell viability. Data normality was confirmed via Shapiro-Wilk tests (all *P* > 0.05), but Mauchly’s Test of Sphericity indicated significant sphericity violation (W = 0.181, *P* < 0.001). Thus, Greenhouse-Geisser corrected results were used for inferential statistics. A highly significant main effect of time was observed (F = 603.843, *P* < 0.001, partial η^2^ = 0.968), demonstrating significant temporal dynamics in cell viability. Additionally, concentration also exerted a highly significant main effect (F = 170.657, *P* < 0.001, partial η^2^ = 0.972), indicating dose-dependent responses to LPS exposure. A significant time-concentration interaction emerged (F = 42.809, *P* < 0.001, partial η^2^ = 0.895). Specifically, significant concentration-dependent differences in cell viability were observed for 6, 12, 24 h (*P* < 0.05), with maximal intergroup divergence at 24 h. However, non-significant intergroup differences (*P* > 0.05), suggesting attenuation of LPS concentration effects with prolonged exposure at 48 h (Figure 2B). Therefore, 24 h of induction was considered optimal. To confirm the optimal LPS dose, RAECs were treated with 0.01, 0.1, 1, and 10 μg/mL LPS for 24 h to investigate whether LPS led to endothelial dysfunction and inflammation. It was found that RAECs treated with 1 μg/mL LPS showed lower NO fluorescence intensity and increased levels of ET-1 as compared to the control group (Figures 3A–C). Furthermore, the contents of TNF-α, MCP-1, IL-1β, and VCAM-1, but not IL-6, were increased in the supernatants compared to the control group (Figures 3D–H). These findings indicated that treatment with 1 μg/mL LPS for 24 h was optimal for induction of endothelial dysfunction and inflammation.

### 3.2 Morphological characteristics and immunofluorescence of hippocampal neurons and cortical microglia

**FIGURE 4:**
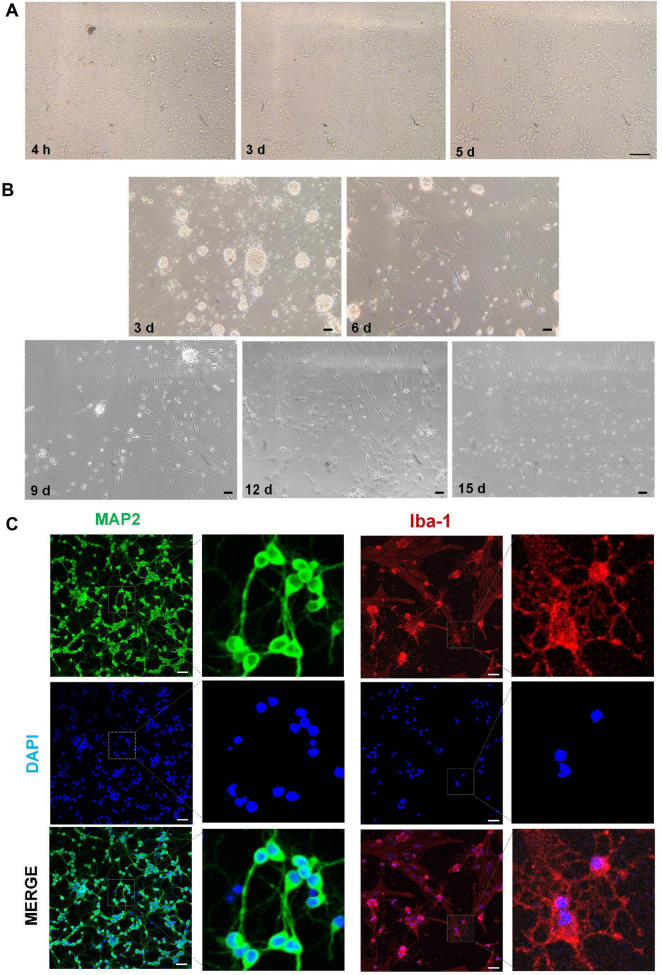
Morphological characteristics and immunofluorescence of hippocampal neurons and cortical microglia. **(A)** Morphological characteristics of hippocampal neurons at different times. **(B)** Morphological characteristics of cortical microglia at different times. **(C)** Immunofluorescence images of neurons (MAP2, green) and microglia (Iba-1, red). Scale bar = 50 μm.

By day 3, the cell somas of the neurons had become larger with dendrites connected by a neural network; these effects were more marked by day 5 (Figure 4A). The microglia exhibited an overall spider-like appearance, with more abundant synaptic spines after 15 days (Figure 4B). Additionally, the cytoplasm of both neurons and microglia was, respectively, positive for MAP2 and Iba-1 (Figure 4C).

### 3.3 Optimization of the tri-culture model of vascular endothelial cells, neurons, and microglia

**FIGURE 5:**
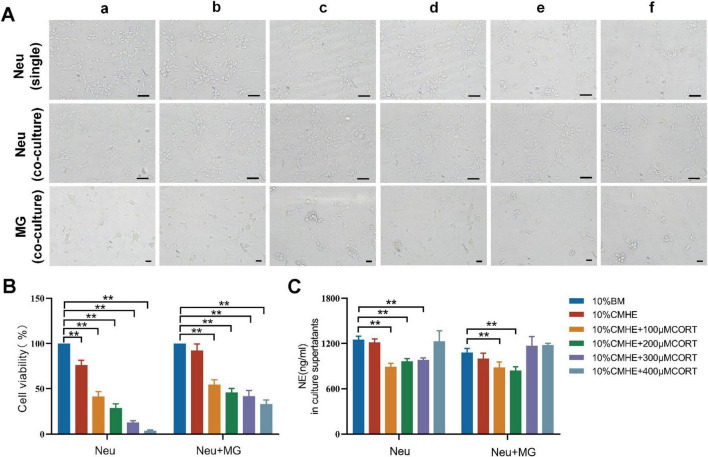
Effects of 10% CMHE combined with different concentrations of CORT on mono- and co-cultures. **(A)** Changes in morphology in the different groups. **(B)** Changes in cell viability in the different groups. **(C)** NE levels in culture supernatants. Data are presented as the mean ± SD (*n* = 3). *P < 0.05, **P < 0.01 vs. 10% BM group (one-way ANOVA followed by LSD or Tamhane’s T2). Scale bar = 50 μm. a: 10% BM group; b: 10% CHME group; c: 10% CHME + 100 μM CORT group; d: 10% CHME + 200 μM CORT group; e:10% CHME + 300 μM CORT group; f:10% CHME + 400 μM CORT group; Neu: neuron; MG: microglia; BM: blank media; CHME: conditioned media of hypertensive environment; CORT: corticosterone; NE: norepinephrine.

After the addition of 10% and 20% concentrations of CMHE to the neurons for 24 h, the survival rates observed were 92.43% and 91.49%, respectively. As these rates did not differ significantly, the 10% concentration of CMHE was selected for further analysis. The conditioned medium served as a simulated hypertensive environment. It was also found that the addition of 10% blank culture medium enhanced neuronal viability, which led to the selection of 10% blank culture medium as the control. In the following experiments, a 10% blank medium was utilized as a control, while a 10% CMHE was selected in combination with varying concentrations of CORT (100, 200, 300, 400 μM) to treat the neuron-microglial co-culture. The levels of NE in the supernatant were assessed. Replicate experiments using neuronal cultures only were performed. Under the same induction conditions, the richness of the neural network and the viability of each neuron group cultured in isolation were found to be lower than those cultured with microglia (Figures 5A, B). The exchange of information between microglia and neurons may enhance neuronal survival. However, regardless of whether neurons were cultured in isolation or in co-culture, the application of 10% CMHE together with varying concentrations of CORT resulted in a decrease in NE content, followed by an increase as the CORT concentration rose. This phenomenon could be attributed to extensive neuronal damage, leading to a significant passive release of NE from vesicles. In alignment with the monoamine neurotransmitter explanation of depression, the combination of 10% CMHE with 200 μM CORT was selected for use in the HD model *in vitro* (Figure 5C).

### 3.4 Cellular injuries in the tri-culture model are induced by the combination of 10% CMHE and 200 μM CORT

**FIGURE 6:**
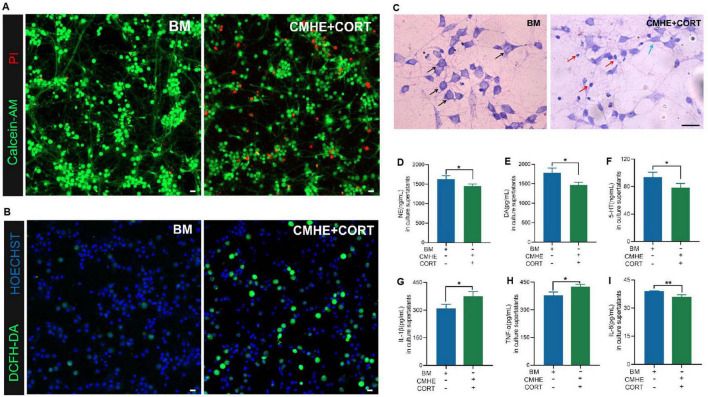
Cellular damage in the tri-culture model induced by combining 10% CMHE with 200 μM CORT. **(A)** Fluorescence images showing the live and dead neurons (PI, red, dead cells; Calcein-AM, green, live cells). **(B)** Fluorescence images of ROS (Hoechst, blue, nucleus; DCFH-DA, green, levels of ROS). **(C)** Images of neuronal Nissl staining (black arrows, abundant Nissl bodies; blue arrows, wrinkled neurons; red arrows, neurons with dissolved Nissl bodies). **(D)** NE levels in culture superntants. **(E)** DA levels in culture supernatants. **(F)** 5-HT levels in culture supernatants. **(G)** IL-1β levels in culture supernatants. **(H)** TNF-α levels in culture supernatants. **(I)** IL-6 levels in culture supernatants. Data are presented as the mean ± SD (*n* = 3). *P < 0.05, **P < 0.01 vs. BM group (T test for two groups). Scale bar = 20 μm. BM: blank media; CHME: conditioned media of hypertensive environment; CORT: corticosterone; NE: norepinephrine; ROS: reactive oxygen species; DA: dopamine; 5-HT: 5-hydroxy tryptamine; IL-1β: interleukin-1beta; TNF-α: tumor necrsis factor alpha; IL-6: interleukin-6.

To clarify whether the conditions used in the previous experiments caused more specific injuries in the model, the structural characteristics, viability, oxidative stress response, secretory functions, and inflammatory levels in cells in the co-culture system were assessed. It was found that the tri-culture model group, compared to the control group, had increased numbers of dead neurons (Figure 6A), elevated levels of ROS (Figure 6B), and more shrunken neurons and dissolved Nissl bodies (Figure 6C). Furthermore, the expression of the monoamine neurotransmitters, NE, DA, and 5-HT, in the supernatant was decreased (Figures 6D–F). In terms of inflammatory factors, the levels of IL-1β and TNF-α were increased, although that of IL-6 was not (Figures 6G–I). These results suggested that the combination of 10% CMHE with 200 μM CORT contributed to a more reliable model of HD.

### 3.5 Effect of TLR4 inhibitor on the tri-culture model in hypertension-related depression

**FIGURE 7:**
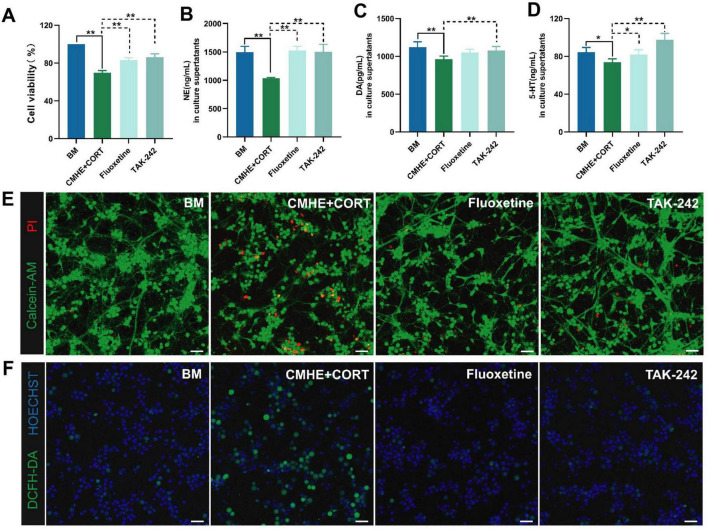
TLR4 inhibitors ameliorate neuronal damage by promoting monoamine neurotransmitter expression and decreasing ROS. **(A)** Changes in cell viability in the different groups. **(B)** NE levels in culture supernatants. **(C)** DA levels in culture supernatants. **(D)** 5-HT levels in culture supernatants. **(E)** Fluorescence images of live and dead neurons (PI, red, dead cells; Calcein-AM, green, live cells). **(F)** Fluorescence images of ROS (Hoechst, blue, nucleus; DCFH-DA, green, expression of ROS). Data are presented as the mean ± SD (*n* = 3). *P < 0.05, **P < 0.01 vs. BM group (one-way ANOVA followed by LSD or Tamhane’s T2). Scale bar = 50 μm. BM: blank media; CHME: conditioned media of hypertensive environment; CORT: corticosterone; ROS: reactive oxygen species.

To determine whether the damage observed in the model was caused by activation of TLR4 signaling pathways, the TLR4 inhibitor, TAK-242, was used with fluoxetine serving as the positive control to assess neuronal injury in the environment of hypertension-related depression. The results showed that treatment with the TLR4 inhibitor enhanced neuronal survival (Figures 7A, E) and reduced ROS generation (Figure 7F). Additionally, increased levels of the monoamine neurotransmitters, NE, DA, and 5-HT, were observed (Figures 7B–D). These changes were similar to the observations of the fluoxetine group, except that the level of 5-HT was higher. Thus, these results suggested that the cell injuries observed in the model may be associated with TLR4 signaling pathways.

**FIGURE 8:**
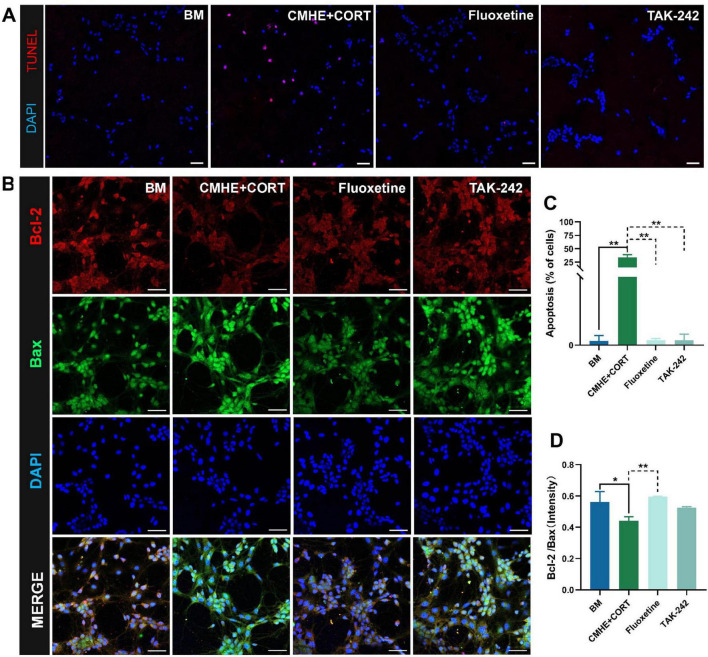
TLR4 inhibitors suppress neuronal apoptosis. **(A)** Fluorescence images of TUNEL staining in the different groups (red, apoptotic cells; blue, nucleus). **(B)** The number of apoptotic cells in the different groups. **(C)** Fluorescence images of apoptosis-related proteins Bcl-2 amd Bax in the different groups. **(D)** Ratio of the relative fluorescence intensity between Bcl-2 and Bax proteins in the different groups. Data are presented as the mean ± SD (*n* = 3). *P < 0.05, **P < 0.01 vs. BM group (one-way ANOVA followed by LSD or Tamhane’s T2). Scale bar = 50 μm. BM: blank media; CHME: conditioned media of hypertensive environment; CORT: corticosterone; Bcl-2: B-Cell Lymphoma 2; Bax: Bcl 2-Associated X.

However, neuronal death can occur through various means, such as ferroptosis, apoptosis, and pyroptosis. TLR4 signaling is known to contribute to neuronal apoptosis (Luo et al., 2023). Thus, neuronal apoptosis in the tri-culture model was assessed using TUNEL fluorescence staining and immunofluorescence. This showed that cells in the tri-culture model had higher levels of apoptosis and lower Bcl-2/Bax ratios, while treatment with TAK-242 reduced apoptosis and increased the Bcl-2/Bax ratio (Figures 8A–D). These findings indicated that blocking TLR4 signaling may contribute to neuronal viability.

**FIGURE 9:**
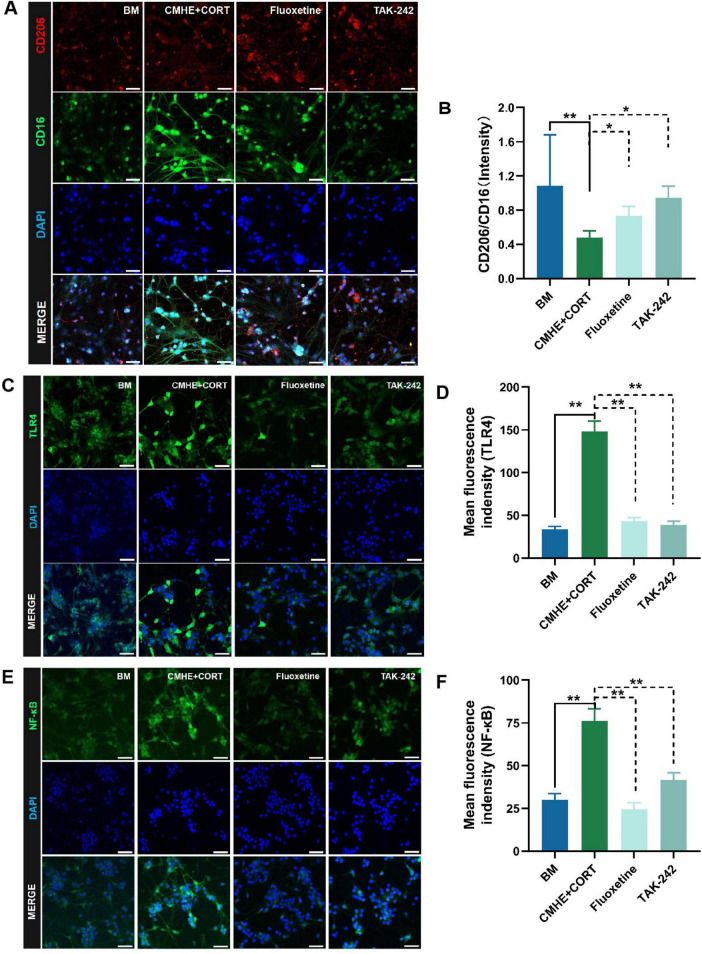
Activation of TLR4/NF-κB signaling and increased microglial M1-type polarization induces neuronal damage in the tri-culture model. **(A)** Fluorescence images of CD206 amd CD16 proteins in the microglia in the different groups. **(B)** Ratio of the relative fluorescence intensities between CD206 amd CD16 proteins in microglia. **(C)** Fluorescence images of TLR4 protein in neurons in the different groups. **(D)** Changes in fluorescence intensity of TLR4 protein in neurons in the different groups. **(E)** Fluorescence images of NF-κB protein in neurons in the different groups. **(F)** Changes in fluorescence intensity of NF-κB in neurons in the different groups. Data are presented as the mean ± SD (*n* = 3). *P < 0.05, **P < 0.01 vs. BM group (one-way ANOVA followed by LSD or Tamhane’s T2). Scale bar = 50 μm. BM: blank media; CHME: conditioned media of hypertensive environment; CORT: corticosterone; TLR4: toll-like receptor 4; NF-κB: nuclear factor-κB.

Activation of TLR4 signaling is known to regulate downstream signaling pathways, including the NF-κB and PI3K pathways, inducing inflammation (Hao et al., 2024; Yi et al., 2024). Our previous study suggested that TLR4/NF-κB signaling was activated in the hippocampi of HD rats, leading to polarization of the microglia toward the M1 proinflammatory phenotype. Here, the levels of TLR4, NF-κB, CD16, and CD206 in the tri-culture model were evaluated using immunofluorescence. The results showed that the levels of TLR4 and NF-κB were higher, while the CD16/CD206 ratio was reduced, in microglia from the tri-culture model, and that treatment with TAK-242 reversed these changes (Figures 9A–F). These findings are consistent with previous results in HD rats. It was thus concluded that damage to hippocampal neurons in the tri-culture model was induced by the combination of 10% CMHE and 200 μM CORT through activation of TLR4/NF-κB signaling and promoting microglial M1-type polarization.

## 4 Discussion

Here, the tri-culture model exhibited pathological features that closely resemble those observed in animal models of HD, and we conducted a comparative analysis based on the following aspects. First, spontaneous hypertensive rat models show reduced NO levels, elevated ET-1, inflammatory markers (e.g., IL-1β, TNF-α, MCP-1, VCAM-1), and ROS, indicating endothelial dysfunction, inflammation, and oxidative stress (Chen et al., 2020; Lin et al., 2020; Kuganathan et al., 2024; Wojtacha et al., 2024). In our study, LPS-induced RAECs exhibited similar changes, effectively mimicking endothelial inflammatory damage under hypertensive conditions. Secondly, additional pathological manifestations were observed in HD models (Chen et al., 2023; Ma et al., 2024; Zhao et al., 2024; Zhu et al., 2024): (a) elevated blood pressure levels (exceeding 130 mmHg) and depressive-like behaviors, including behavioral despair and anhedonia. (b) neurons displayed disorganized arrangement, cellular atrophy, increased apoptosis, extensive dissolution of Nissl bodies, and ultrastructural damage including blurred synaptic structures, swollen and fragmented mitochondrial cristae, and reduced synaptic vesicles in the hippocampal tissue. (c) elevated levels of inflammatory factors, including IL-1β, IL-18, TNF-α, COX-2, and MCP-1, alongside reduced secretion of monoamine neurotransmitters such as 5-HT, DA, and NE. (d) activated the TLR4/NF-κB signaling pathway and polarized microglia toward the M1 phenotype in the hippocampus. These findings collectively indicate that HD rats exhibit key pathological features, including high blood pressure, behavioral deficits, hippocampal damage, monoamine neurotransmitter dysregulation, and neuroinflammation. In our tri-culture model, neurons exhibited reduced viability, increased apoptosis, decreased Nissl bodies, elevated levels of IL-1β and TNF-α, and reduced levels of monoamine neurotransmitters such as 5-HT, DA, and NE. Furthermore, a significant increase in M1-polarized microglia was observed, accompanied by elevated protein levels of TLR4 and NF-κB. These findings closely resemble the pathological manifestations observed in the HD rat, including endothelial dysfunction, inflammation, monoamine neurotransmitter deficiency, damage to hippocampal neurons, and microglial activation.

Hypertension is generally considered a chronic low-grade vascular inflammation. The pathophysiological mechanism is closely associated with vascular endothelial function (Behnoush et al., 2023; Tomiyama, 2023) and ameliorating vascular endothelial dysfunction is an effective antihypertensive treatment (Pang et al., 2023; Bian et al., 2024). Hypertension increases shear stress, damaging endothelial cells and triggering inflammation via cytokines (e.g., MCP-1, IL-1β, TNF-α) (D’Onofrio et al., 2023). Reduced NO production and elevated vasoconstrictors (e.g., Ang II, ET-1) exacerbate endothelial dysfunction (Yang et al., 2024). Decreased claudin-5 and increased caveolin-1 disrupt the blood-brain barrier (BBB) (Atis et al., 2022). Inflammatory factors secreted by vascular endothelial cells enter the brain from the circulation through the BBB, leading to cytokine production and monocyte activation. This, in turn, leads to microglial activation and the production of proinflammatory factors at the expense of neurotrophic factors, contributing to depression associated with dysfunctional neurotransmitter metabolism, disordered glutamatergic transmission, and altered synaptic plasticity (Miller and Raison, 2016; Bruno et al., 2020; Dixon et al., 2020; Chu et al., 2021). This may be why patients with hypertension are more susceptible to depression (Li et al., 2015). Endothelial cells from the brain microvascular endothelium, aorta, and human umbilical veins, among others, have been used in the study of cardiovascular diseases (Shen et al., 2015; Shen et al., 2020; Park et al., 2022; Yang et al., 2022).

The pathogenesis of HD is complex, and most studies have focused on immune inflammation, especially hippocampal damage caused by cerebral inflammation. The norepinephrine transporter is involved in hypertension and depression by regulating TNF-α and IL-6 (Meng et al., 2020). TLR4 activation promotes the inflammation-related feed-forward pro-hypertensive cycle (Mowry et al., 2021). TLR4/NF-κB signaling has been found to mediate inflammation in the hippocampus and aggravate depressive-like behavior in rats (He et al., 2021). TLR4 binding to appropriate ligands activates the microglia and induces the production of proinflammatory cytokines, while blocking TLR4 reduces microglial activation (Xiong et al., 2025). In this study, a greater number of M1-type microglia and higher levels of both TLR4 and NF-κB were found in the tri-culture model. Neurons are the cells responsible for signal transmission, while the microglia, the immune cells in the cerebrum, contribute to neuronal survival. The interactions between the two cell types have been extensively investigated due to their impact on neurological diseases (Chen and Bu, 2024). Co-culture models comprising neurons and microglia have been widely used for research on neuroinflammation (Grubman et al., 2020). However, there have been no studies on co-culture models for HD, making research on HD pathogenesis more challenging. Therefore, the construction of a composite tri-culture model consisting of endothelial cells, neurons, and microglia that can simulate the HD environment is key to the investigation of hypertension-related depression diseases.

Lipopolysaccharide activates TLR4, triggering NF-κB signaling, upregulating pro-inflammatory mediators, and inducing endothelial damage via apoptosis, autophagy, and increased permeability (Wu et al., 2022). LPS also activates COX-2, producing PGE2, which disrupts the BBB (Kikuchi et al., 2019). To create a microenvironment similar to that of depression associated with hypertension, RAECs were treated with LPS to establish a cell model of the hypertensive inflammatory microenvironment. LPS is an effective stimulant of inflammation that can activate endothelial cells and promote the release of proinflammatory cytokines (Song et al., 2021). Although the LPS-induced RAEC injury model has been used to study the mechanism of cardiovascular disease, there is limited information on the specific conditions required for induction, such as concentrations and times, due to a lack of quantitative standards and evaluation indicators (Hu et al., 2021). Therefore, we first treated RAECs with 0.01, 0.1, 1, and 10 μg/ml LPS for 6, 12, 24, and 48 h to evaluate cell viability, endothelial function, and inflammatory factor levels to determine the optimal concentrations and times. This showed that treatment with 1 μg/ml of LPS for 24 h resulted in reduced levels of intracellular NO, accompanied by increases in secreted ET-1 and the levels of the inflammatory factors TNF-α, MCP-1, IL-1β, and VCAM-1. These results are consistent with those of previous studies (Gutiérrez et al., 2023; Liu et al., 2023; Costa et al., 2024), including the reduced levels of eNOS and NO, changes in the ET-1 levels, and increases in proinflammatory factors in dysfunctional endothelial cells. NO and ET-1 are respective indicators of relaxation and contraction in vascular endothelial cells. Drugs such as cyclooxygenase 2 inhibitors and inhibitors of the renin-angiotensin system can alleviate endothelial dysfunction by targeting endothelial inflammation (Wang et al., 2022).

Here, culture supernatants of RAECs treated with 1 μg/mL LPS were used for CMHE, and the supernatant without LPS was used for BM. It was found that both 10% and 20% BM increased the viability of hippocampal neurons in a dose-dependent manner, while both 10% and 20% CMHE reduced neuronal viability although the difference was not significant. The reason may be that the conditioned medium promotes intercellular communication through paracrine factors. Conditioned media from microglia have been shown to protect neurons from ferroptosis (Jacques et al., 2023). BM contains secreted factors that benefit neuronal survival, while the presence of various proinflammatory factors in CMHE inhibited neuronal survival. Therefore, the establishment of a tri-culture model using 10% CMHE combined with CORT to interfere with neuron-microglia interactions should use 10% BM as a blank control.

Corticosterone, a glucocorticoid, induces depression-like effects by inhibiting tryptophan hydroxylase, altering dopaminergic neuron activity, reducing dendritic spine density in the hippocampus and prefrontal cortex, activating microglia, and increasing ROS production. These changes disrupt neurotransmitter systems, neuroplasticity, and promote neuroinflammation and oxidative stress (Choi et al., 2021; Sun et al., 2021; Dang et al., 2024; Shi et al., 2024; Ru-han et al., 2025). Primary hippocampal neurons treated with CORT are frequently used in studies on depression and damage to hippocampal neurons (Zhang et al., 2023; Wang et al., 2020). The present study found that neuronal viability was higher in the co-culture group compared to the single group when using the same CORT concentration. Intriguingly, as the CORT concentration increased, whether in single culture or the co-culture system, the NE contents initially decreased and then increased, respectively, at CORT concentrations of 100 and 200 μM. This may have been due to extensive neuronal damage and dysfunctional reuptake, resulting in the release of NE from vesicles. However, the presence of the microglia was beneficial to maintaining neuronal viability and regulating the levels of neurotransmitters, consistent with the findings of previous studies (Jacques et al., 2023).

The TLR4/NF-κB axis is a classic pro-inflammatory signaling pathway, and many studies (Qi Q. et al., 2024; Qi X. et al., 2024) have demonstrated a relationship between the pathway and apoptosis. The present study measured apoptosis with TUNEL assays and the levels of the apoptosis-related proteins Bcl-2 and Bax. It was found that the neurons in the model group had higher expression of TLR4 and NF-κB, more M1-type microglia, increased inflammation, and more apoptotic neurons compared to the normal group. Thus, activation of the TLR4/NF-κB pathway may aggravate inflammation by promoting microglial polarization, ultimately leading to neuronal apoptosis in the tri-culture model. Intriguingly, these findings are consistent with those of our previous studies on HD rats (Zhao et al., 2024; Ma et al., 2024) that observed morphological damage to neurons, activation of microglia, activated TLR4/NF-κB signaling, and increased expression of Bcl-2.

In conclusion, we successfully established an *in vitro* tri-culture model consisting of rat aortic endothelial cells, neurons, and microglia using a combination of conditioned medium and a modification of the Banker co-culture method. This model was more similar to physiological conditions and simulated intercellular communication better compared to single-cell cultures. Additionally, the integration of factors used in the model may make it more effective and simulate the complex pathogenesis of hypertension-related depression compared to a single inducer. The findings may provide an experimental foundation for studying the mechanisms of HD and other neurological diseases. However, this model has several limitations, including lack of in-depth investigation of model stability and different times of induction. In future studies, it’ s key to access the long-term stability of the model through time-course experiments, monitoring cellular interactions and signaling dynamics (e.g., TLR4, NF-κB, NLRP3, CD16, CD206, Bcl-2, Bax) using immunofluorescence and Western blot. Key functional markers, including VE-Cadherin, ZO-1, Occludin (endothelial cells), MAP2, VGLUT1 (neurons), and Iba-1, CD86, CD206, NF-κB, TREM2 (microglia), will be quantified to evaluate cellular functionality and model stability over time. Additionally, incorporating long-term exposure experiments (e.g., 7–14 days) would better mimic the temporal dynamics of cellular and molecular changes, such as sustained inflammation, oxidative stress, and neuronal damage, which are hallmarks of chronic HD.

## Data Availability

The original contributions presented in the study are included in the article/Supplementary material, further inquiries can be directed to the corresponding author.
