# Supplementary material for: A vascular endothelial cell, neuron, and microglia tri-culture model to study hypertension-related depression
Source: Front Cell Neurosci. 2025 Mar 31;19:1553309. doi: 10.3389/fncel.2025.1553309 (PMC11994666; doi:10.3389/fncel.2025.1553309)
Supplement: Supplementary file 1 [file Data_Sheet_1.pdf]

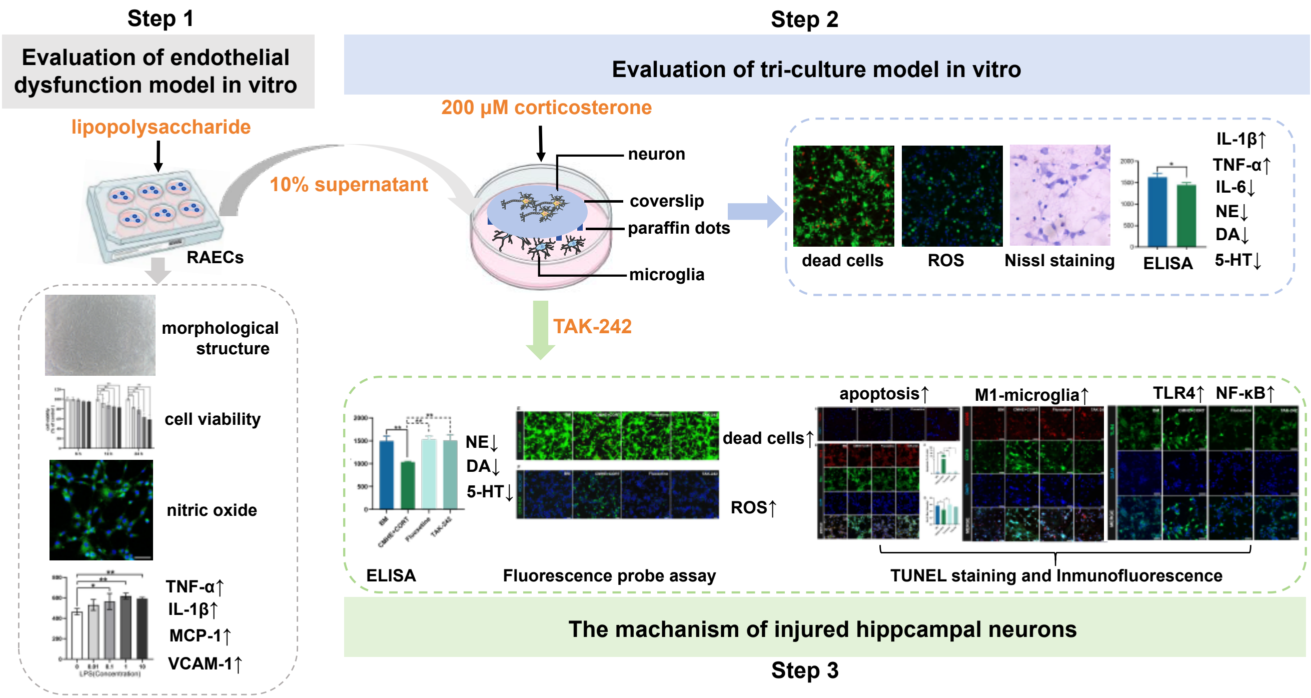
Step 1
Evaluation of endothelial dysfunction model in vitro
lipopolysaccharide
RAECs
10% supernatant
morphological structure
cell viability
nitric oxide
TNF-α↑
IL-1β↑
MCP-1↑
VCAM-1↑
Step 2
Evaluation of tri-culture model in vitro
200 μM corticosterone
neuron
coverslip
paraffin dots
microglia
dead cells
ROS
Nissl staining
ELISA
IL-1β↑
TNF-α↑
IL-6↓
NE↓
DA↓
5-HT↓
TAK-242
NE↓
DA↓
5-HT↓
dead cells↑
ROS↑
apoptosis↑
M1-microglia↑
TLR4↑
NF-κB↑
ELISA
Fluorescence probe assay
TUNEL staining and Inmunofluorescence
The machanism of injured hippcampal neurons
Step 3
